# Characterizing the Neuroimmune Environment of Offspring in a Novel Model of Maternal Allergic Asthma and Particulate Matter Exposure

**DOI:** 10.21203/rs.3.rs-3140415/v1

**Published:** 2023-07-10

**Authors:** Juan M. Tamayo, Hadley C. Osman, Jared J. Schwartzer, Kent Pinkerton, Paul Ashwood

**Affiliations:** University of California at Davis; University of California at Davis; Mount Holyoke College; University of California at Davis; University of California at Davis

**Keywords:** Neurodevelopment, Cytokines, Autism spectrum disorder (ASD), Schizophrenia, Neuroinflammation, Asthma/Allergy, Fetal brain

## Abstract

Autism spectrum disorders (ASD) are neurodevelopmental disorders characterized by the presence of decreased social interactions and an increase in stereotyped and repetitive behaviors. Epidemiology studies suggest that cases of ASD are on the rise. Similarly, rates of asthma are increasing, and the presence of maternal asthma during pregnancy increases the likelihood of a child being later diagnosed with ASD. Particulate matter (PM), via air pollution, is an environmental factor known to worsen the symptoms of asthma, but also, PM has been associated with increased risk of neuropsychiatric disorders including ASD. Despite the links between asthma and PM with neuropsychiatric disorders, there is a lack of laboratory models investigating combined prenatal exposure to asthma and PM on offspring neurodevelopment. Thus, we developed a novel mouse model that combines exposure to maternal allergic asthma (MAA) and ultrafine iron-soot (UIS), a common component of PM. In the current study, female BALB/c mice were primed for allergic asthma with ovalbumin (OVA) prior to pregnancy. Following mating and beginning on gestational day 2 (GD2), dams were exposed to either aerosolized OVA or phosphate buffered saline (PBS) for 1 hour. Following the 1-hour exposure, pregnant females were then exposed to UIS or clean air for 4 hours. Offspring brains were collected at postnatal days (P)15 and (P)35. Cortices and hippocampal regions were then isolated and assessed for changes in cytokines using a Luminex bead-based multiplex assay. Analyses identified changes in many cytokines across treatment groups at both timepoints in the cortex, including interleukin-1 beta (IL-1β), IL-2, IL-13, and IL-17, which remained elevated from P15 to P35 in all treatment conditions compared to controls. In the hippocampus at P15, elevations in cytokines were also identified across the treatment groups, namely interferon gamma (IFNγ) and IL-7. The combination of MAA and UIS exposure (MAA-UIS) during pregnancy resulted in an increase in microglia density in the hippocampus of offspring, as identified by IBA-1 staining. Together, these data indicate that exposure to MAA, UIS, and MAA-UIS result in changes in the neuroimmune environment of offspring that persist into adulthood.

## 1. BACKGROUND

Autism spectrum disorders (ASD) are neurodevelopmental disorders characterized by the presence of decreased social interactions and an increase in stereotyped and repetitive behaviors. Epidemiology studies suggest that cases of ASD are on the rise, with 1 in 44 children born in the United States later being diagnosed with ASD (Maenner et al., 2021). There does not appear to be a single cause for ASD, but research has shown that the etiology of some cases can be connected to heritable susceptibility from both genetics and the environmental exposures in utero. Depending on the study, genetic heritability can account for more than 50% of cases. However, having the susceptibility genes does not guarantee that a child will be diagnosed with ASD. In other words, many of the associated risk genes for ASD are common genetic variants in the population and carry a low risk (Gaugler et al., 2014). Given that genetic risk factors have only a moderate contribution to the observed cases of ASD (Devlin et al., 2011; Klei et al., 2012), it is important to identify environmental risk factors that can contribute to ASD incidence. Mounting clinical and epidemiological data underscore the notion that environmental factors play a large role in both the underlying development of ASD and their impact on the severity of individual behavioral characteristics (Hughes et al., 2018; Rossignol et al., 2014; Straughen et al., 2021; Raz et al., 2015; Volk et al., 2011; Volk et al., 2020; Patterson, 2009). Pregnancy, in particular, marks a critical period when environmental insults have long lasting effects on neurodevelopment.

The maternal immune system represents a key biological mechanism that links environmental factors to specific neurodevelopmental changes and later ASD diagnosis. For example, clinical studies have linked hospitalizations for bacterial and viral infections during pregnancy to an increased rate of birthing a child that will later be diagnosed with ASD (Modabbernia et al., 2017; Tioleco et al., 2021; Atladóttir et al., 2010; Brown et al., 2014). Moreover, laboratories have developed animal models that often use bacterial or viral mimics, such as lipopolysaccharide (LPS) or polyinosinic:polycytidylic acid (Poly(I:C)), respectively, to induce maternal inflammation during pregnancy and demonstrate a causal change in offspring brain and behavioral health (Han et al., 2021). Despite the efficacy of these models in demonstrating a link between maternal immune signaling and offspring neurodevelopment, they are limited in representing many of the common environmental sources of immune activation. With perhaps the exception of SARS-CoV-2, hospitalizations for viral infections are less frequent, and a recent meta-analysis of viral infections during pregnancy as a risk for children later being diagnosed with ASD was not supported (Jiang et al., 2016). Importantly, infections via bacterial and viral pathogens are only two of many ways in which the immune system can become activated, and researchers have begun to investigate other common environmental influences that can have a substantial impact on the immune system and offspring brain development, most notably asthma.

Rates of asthma, like ASD, are currently increasing and represent a highly prevalent chronic disease that more commonly impacts ethnic/racial minority and socioeconomically disadvantaged groups in the US (Clougherty et al., 2007; Matsui et al., 2008). Importantly, chances of asthma exacerbations increase during pregnancy, and the presence of asthma increases likelihood of birthing a child that is later diagnosed with ASD (Ali & Ulrik, 2013; Murphy, et al., 2005; Murphy et al., 2006; Croen et al., 2005; Croen et al., 2019; Gong et al., 2019; Abdallah et al., 2011; Hisle-Gorman et al., 2018; Lyall et al., 2014; Patel et al., 2020; Fasmer et al., 2011). Asthma is an inflammatory disease of the lungs characterized by bronchial hyperresponsiveness, persistent inflammation, airflow obstruction, and reduction of airflow (Gluck & Gluck, 2006; Popa et al., 2021). The allergens associated with asthma can vary and include, but are not limited to rodent allergen, cockroach allergen, pollen, and house dust mite (Matsui et al., 2008; Jacquet 2013). Given the incidence of allergies and asthma in the United States (Blackwell et al., 2014; Fazel et al., 2018) and the high prevalence of allergic triggers in urban environments (Leaderer et al., 2002; Matsui et al., 2008; Salo et al., 2008; Claeson et al., 2016), there is pressing need to better understand the causal effects of asthma during pregnancy and the impact of specific allergic triggers on offspring mental health.

Using our mouse model of maternal allergic asthma (MAA) to initiate an immune response in pregnant mice, we have previously reported systemic elevations in interleukin-4 (IL-4) and IL-5 in dams (Schwartzer et al., 2015; Tamayo et al., 2022) that parallel clinical reports associating increased IL-4 and IL-5 in mid-pregnancy maternal serum samples of mothers with children later diagnosed with ASD (Goines et al 2011). Not only do the dams in our MAA model produce a similar allergic asthma cytokine profile to that observed in humans, but the offspring display characteristic ASD-like behaviors, such as decreased social interaction and increased repetitive-like behaviors (Schwartzer et al., 2015; Schwartzer et al., 2017; Church et al., 2021). Moreover, MAA produces transcriptional differences in microglia gene expression and neuroinflammation in both prenatal offspring and brain regions of adult mice (Ciernia et al., 2018; Church et al., 2021; Tamayo et al., 2022). These findings highlight the lasting changes that allergic asthma during pregnancy can have on offspring neurodevelopment. However, environmental insults associated with ASD can vary, and exposure to these environmental factors do not necessarily occur in isolation for humans. In fact, individuals often simultaneously encounter multiple inflammatory-inducing environmental stimulants, and less is known about the potential synergistic effects of these exposures on maternal immune response and subsequent offspring development (Leaderer et al., 2002; Salo et al., 2008; Claeson et al., 2016).

Particulate matter (PM), via air pollution, is another environmental factor that is suspected to be associated with the risk of neuropsychiatric disorders such as schizophrenia, attention deficit hyperactive disorder (ADHD), and ASD (Volk et al., 2011; Volk et al., 2013; Volk et al., 2020). Not only is PM linked to neurodevelopmental disruptions when exposures occur during pregnancy (Church et al 2018), but it has also been tied to exacerbated asthma responses and could potentially cause new onset cases of asthma (Guarnieri & Balmes, 2014). This is especially true for ultrafine PM (PM0.1; aerodynamic diameter < 0.1 μm) (Chan et al., 2011; Schraufnagel 2020; Chalupa et al., 2004). PM is a complex mixture of constituents that contains organic compounds, soot, metals and metal-oxides, nitrates, and other elements in varying quantities depending on its source (Steiner et al., 2016). As such, it is necessary to characterize individual components of PM in order to effectively regulate air quality for limiting environmental exposure to toxicants.

Diesel exhaust is one common source of PM that has been identified as a risk factor for ASD (Kalkbrenner et al., 2015; Volk et al., 2011; Volk et al., 2013; Volk et al., 2020; von Ehrenstein et al., 2014; Roberts et al., 2013). Combustion-derived diesel exhaust includes soot particles containing elemental carbon and iron, which is the most common transition metal found in PM (Zhou et al., 2003). Iron in PM can occur from fuel additives and as a product of normal engine wear (Mayer et al., 2010; Steiner et al., 2016), and iron-soot (IS) exposure has previously been shown to cause oxidative lung injury, induce inflammation of the lungs, and can cross the blood brain barrier through nasal inhalation (Zhong et al., 2010; Zhou et al., 2003; Hopkins et al., 2018). These links to worsening of asthma symptoms and increased risk of neuropsychiatric disorders in offspring make PM exposure during pregnancy an important area that merits investigation.

There are many human studies and animal models investigating the links between gestational exposure to PM and neurodevelopmental outcomes in offspring (Volk et al., 2011; Volk et al., 2013; Volk et al., 2020; Bolton et al., 2017; Ahadullah et al., 2021). In addition, many studies have investigated the impacts of asthma as a risk factor for ASD, including our own (Schwartzer et al., 2015; Schwartzer et al., 2017; Vogel Ciernia et al., 2018; Church et al., 2021; Tamayo et al., 2022; Ali & Ulrik, 2013; Murphy, et al., 2005; Murphy et al., 2006; Croen et al., 2005; Croen et al., 2019; Gong et al., 2019; Abdallah et al., 2011; Hisle-Gorman et al., 2018; Lyall et al., 2013; Patel et al., 2020; Fasmer et al., 2011). However, these investigations consider asthma or PM only as independent risk factors for developing neuropsychiatric disorders. Despite the links between allergic asthma with ASD and the link between PM and ASD, there are no studies investigating the neuroimmune outcome on offspring of these environmental factors in conjunction. This is an apparent oversight given that PM exposure can worsen symptoms of asthma (Guarnieri & Balmes, 2014), and the source of environmental factors contributing to ASD are likely multifaceted.

In this study, we hypothesized that when mice are exposed to MAA and ultrafine iron-soot (UIS) particles during pregnancy (MAA-UIS), the neuroimmune outcomes will show heightened inflammation in offspring compared to that of MAA or UIS exposure alone. We also suspected that, because microglia can respond to changes in the neuroimmune environment through proliferation and are suspected to be associated with ASD behaviors (Hughes et al., 2020; Davoli-Ferreira et al., 2021), we would see signs of functional differences in the frontal cortex and hippocampus–two brain regions implicated as being impacted developmentally in neuropsychiatric disorders such as ASD (Ecker2017; Hughes et al., 2020; Richards et al., 2020; Banker et al., 2021). Using our established mouse paradigm of maternal aerosol exposure to study offspring outcomes, we demonstrate that MAA and UIS alone, as well as MAA and UIS combined, alter the neuroimmune profile in the brains of offspring that is sustained from adolescence into early adulthood.

## 2. METHODS

### 2.1 Animals

BALB/c male and female mice were obtained from breeding pairs originally purchased from Envigo Laboratories (Livermore, CA, USA) and maintained at University of California, Davis at the Center for Health and Environment, Davis, CA. Mice were housed with same-sex littermates and kept at ambient room temperature (23°C) on a 12 h light/dark cycle (lights on at 0800 h) within ventilated cages with water and food provided *ad libitum*. All procedures were performed with approval by University of California Davis Institutional Animal Care and Use Committee and according to guidelines established by National Institute of Health Guide for the Care and Use of Laboratory Animals.

### 2.2 Maternal allergic asthma and ultra-fine soot particle exposure

Female BALB/c (n = 8/group) mice were sensitized with 10 μg of ovalbumin (OVA, Sigma, St. Louis, MO, USA) and 1 mg (Al) OH3 dissolved in 200 μl of phosphate buffered saline (PBS) injected intraperitoneally at 7 and 8 weeks of age. Control dams were injected with PBS alone. Dams were then mated with age matched males and checked daily for the presence of a seminal plug, which was noted as gestational day (GD) 0.5. Beginning on gestational day 2 (G2), dams were exposed to either aerosolized OVA or phosphate buffered saline (PBS) for 1 hour, depending on treatment group. Following the 1-hour exposure, mice were placed in a 20 cm x 43 cm x 18 cm polycarbonate whole-body chamber for exposure to an aerosol of ultrafine iron-soot (target concentration of 200 μg/m^3^), including 40 μg/m^3^ of iron-oxide nanoparticles, or sham control (AIR). The total iron-soot generated was cooled and diluted with filtered air to achieve the desired concentration prior to reaching the exposure chambers. Mice were exposed for 4 hours/day on G2, G4, G6, G9, G11, G13, G16, and G18 to PBS or OVA [MAA condition] and AIR or ultrafine iron-soot [UIS condition], resulting in a total of four treatment groups: PBS/AIR (PBS-AIR), MAA/AIR (MAA), PBS/UIS (UIS), MAA/UIS (MAA-UIS). Following the last day of aerosol exposure (G18) pregnant mice were left undisturbed through parturition. Offspring were either sacrificed at postnatal day (P)15 or weaned at P21, housed with same-sex littermates, and sacrificed at P35 for brain tissue analysis.

### 2.3 Generation and characterization of particles

UIS particles were generated as previously described by Hopkins et al. (2018). Briefly, particle generation was obtained using a laminar diffusion flame system by mixing ethylene gas (the primary hydrocarbon fuel) and acetylene gas to compensate for the effect of iron-oxide to suppress soot formation. By reaching the vapor phase of iron pentacarbonyl by warming to 20°C with combusted argon carrier gas (all Sigma-Aldrich Chemical Co., St. Louis, MO) in the presence of an ethylene/acetylene vapor mix, the source of iron was generated. The result of these combusted reactants generated a hetero-disperse aerosol of ultrafine iron oxide particles (Fe_2_O_3_) and associated soot. Further details of the system and particle generation can be found in previously published studies (Jasinski et al., 2006, Pinkerton et al., 2008; Hopkins et al., 2018). In order to simulate unhealthy air quality conditions, an average particle concentration of 200 μg/m^3^ was selected, as this reflects a concentration that is reminiscent of heavy pollution and poor air quality days in many parts of the world (Pui et al., 2014; Hopkins et al., 2018).

### 2.4 Cardiac perfusion and brain tissue collections

At P15 and P35, offspring were collected from their home cages, anesthetized using isoflurane (2–4% inhalation) and underwent transcardial perfusion. Briefly, a lateral incision was made in the abdominal wall below the rib cage. With curved scissors, an incision was made in the diaphragm and cuts were made along the ribs to the collarbone to allow the sternum to be lifted. Once exposed, the heart was inserted with a 15-gauge perfusion needle into the ascending aorta for entry of perfuse, and an incision was made into the right atrium to create an outlet for drainage. Using a perfusion pump, 20 mL of PBS was slowly pumped through the circulatory system to reach adequate clearing. Whole brains were removed and dissected into hemispheric halves. One half was further dissected into cortical and hippocampal regions, flash frozen with liquid nitrogen, and stored at −80°C for later use in cytokine analyses. The remaining half was placed in 4% PFA for fixation for 24 hours, following this, it was then placed in 30% sucrose for 24 hours for cryoprotection. Cryoprotected tissues were then embedded in optimal cutting temperature (O.C.T.) media and frozen at −80°C.

### 2.5 Tissue sectioning and staining

Frozen tissue embedded in O.C.T. was sectioned with a Leica Instruments cryostat at 20 μm. Free-floating tissue sections were stored in PBS containing 0.01% sodium azide. Sections were incubated in 1:1,000 rabbit-anti IBA-1 (Wako, Neuss, Germany) with 10% normal goat serum (NGS) and 0.2% triton X-100 at 4°C for 24 hours, followed by 1 hour incubation with goat-anti rabbit biotinylated secondary antibody (Vector Laboratories, Burlingame, CA) in 5% NGS for 1 hour at room temperature. Tissue sections were then incubated with avidin-biotinylated HRP complex (Vectastain Elite ABC kit, Vector Laboratories, Burlingame, CA) at room temperature. Visualization of labeling was conducted using 3,3’-diaminobenzidine (DAB) solution in the presence of peroxidase (HRP) enzyme. All sections were thoroughly rinsed three times with 1X PBS between staining steps. Sections were mounted onto glass Superfrost Plus microscope slides and cover slipped with VectaMount Permanent Mounting Medium. Once dry, 20X images were taken on a brightfield microscope and stitched together using Photoshop version 22.3 (Adobe Inc., San Jose, CA, 2023). A macros script in ImageJ version 1.53 (U.S. National Institutes of Health, Bethesda Maryland, 2022) was used to quantify microglia in order to limit user bias.

### 2.6 Stereology

IBA-1 positive microglia were identified using stereological methods. IBA-1 cell counts were made on a brightfield microscope (Nikon Eclipse Ci, Nikon, Tokyo) at 20X magnification, and images were taken using NIS Elements v.4.0 (Nikon Instruments Inc. 1300 Walt Whitman Road Melville, NY 11747 – 3064, U.S.A.). Image analysis was performed using a macros script in ImageJ version 1.53 (U.S. National Institutes of Health, Bethesda Maryland, 2022) to quantify microglia. A total of six to nine sections per brain region were collected. Counts of microglia cells were taken from infralimbic and anterior cingulate cortical areas of the frontal cortex, as well as the dentate gyrus, CA1, CA2, and CA3 of the hippocampus. Microglia were identified by IBA-1 positive cell body staining.

### 2.7 Multiplex Bead-Based Cytokine Analysis

Analysis of serum cytokines was performed using a multiplex mouse 25-plex bead immunoassay (Milliplex Mouse Cytokine/Chemokine Magnetic Bead Panel #MCYTMAG70PMX25BK). The following cytokines were quantified: granulocyte colony stimulating factor (G-CSF), granulocyte colony stimulating factor (GM-CSF), IFN-γ, IL-1α, IL-1β, IL-2, IL-4, IL-5, IL-6, IL-7, IL-9, IL-10, IL-12 (p40), IL-12 (p70), IL-13, IL-15, IL-17, interferon gamma-induced protein 10 (IP-10), keratinocyte chemoattractant (KC), monocyte chemoattractant protein-1 (MCP-1), macrophage inflammatory protein-1 alpha (MIP-1α), MIP-1β, MIP-2, chemokine ligand 5 (RANTES), and tumor necrosis factor alpha (TNF-α). Standards and reagents were all prepared according to the manufacturers’ recommendations. Each brain sample was diluted to a standardized concentration and run in duplicate. Twenty-five microliters of sample, standards, or blanks were loaded into a 96-well plate with appropriate amounts of assay buffer and matrix solution. The plate was then incubated overnight with antibody-coupled magnetic beads. The following day, the plate underwent a series of washes. Washes were performed using a Bio-Plex handheld magnet (Bio-Rad Laboratories, Hercules, CA, USA). After the final wash, the plate was incubated with biotinylated detection antibody on a shaker for 30 min, and analyzed using a Bio-Rad Bio-Plex 200 plate reader (Bio-Rad Laboratories, Hercules, CA, USA). The following were the minimal amounts of detectable cytokine concentration: G-CSF: 1.7 pg/mL; GM-CSF: 10.9 pg/mL; IFNγ: 1.1 pg/mL; IL-1α: 10.3 pg/mL; IL-1β: 5.4 pg/mL; IL-2:1.0 pg/mL; IL-4: 0.4 pg/mL; IL-5:1.0 pg/mL; IL-6:1.1 pg/mL; IL-7:1.4 pg/mL; IL-9:17.3 pg/mL; IL-10: 2.0 pg/mL; IL-12 (p40): 3.9 pg/mL; IL-12 (p70): 4.8 pg/mL; IL-13: 7.8 pg/mL; IL-15: 7.4 pg/mL; IL-17: 0.5 pg/mL; IP-10: 0.8 pg/mL; KC: 2.3 pg/mL; MCP-1: 6.7 pg/mL; MIP-1α: 7.7 pg/mL; MIP-1β: 11.9 pg/mL; MIP-2: 30.6 pg/mL; RANTES: 2.7 pg/mL; TNF-α: 2.3 pg/mL. Sample concentrations that fell below the minimal detection value were given a proxy value of half the limit of detection for statistical comparisons.

### 2.8 Statistical analysis

Brain cytokine data were analyzed using linear-mixed effects modeling to control for the unexplained residual variance that could be originating from litter-to-litter variations due to the hierarchical data structure in which statistical independence of observation is violated (Lazic and Essioux, 2013; Lazic et al., 2018, Brauer and Curtin, 2018; Schielzeth and Nakagawa, 2013; Wainwright et al., 2007). Models were built with the lme package of R version 4.2.2 using a forward-stepwise approach. First, a random-effects only model was constructed with litter set as the random effect. Then fixed effects for treatment (PBS or MAA; AIR or UIS) and sex (male or female) were added followed by a full model containing both treatment, sex, and their interaction. Model fit was assessed using the likelihood ratios test and the best model was selected based on the Akaike Information Criterion (AIC). For models with significant interactions, fixed effects were further analyzed using pairwise comparisons of estimated marginal means.

## 3. Results

### 3.1 P15 offspring cortex cytokines

**Figure 1:**
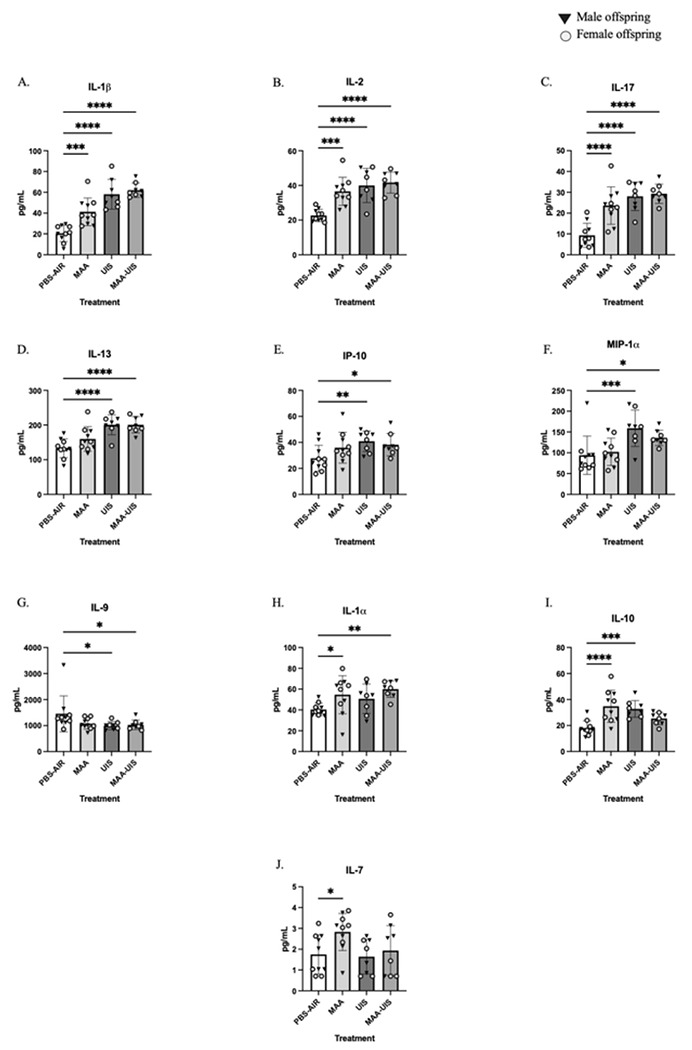
Cortical cytokine concentrations in P15 offspring brains exposed to PBS-AIR, MAA, UIS, or MAAUIS. Cytokines were assessed using multiplex bead-based immunoassays. (A) IL-1β, (B) IL-2, (C) IL-17, (D) IL-13, (E) IP-10, (F) MIP-1α, (G) IL-19, (H) IL-1α, (I) IL-10, and (J) IL7 are represented as pg/mL after being normalized to total protein content. Statistical significance was determined by multilevel mixed-effects modeling.

Offspring brains were collected and micro-dissected into cortical and hippocampal sections at P15. Homogenates of each section were then analyzed for cytokine concentration. Multilevel mixed-effects modeling was used to control for within-litter variability and inclusions of fixed effects was determined using forward stepwise regression. For all cytokines measured, the inclusion of sex-difference did not significantly improve model fit (Supplementary Table Fig. 1). In the cortex, several cytokines were elevated in both male and female offspring of MAA, UIS, and MAA-UIS dams, many of which are generally considered inflammatory and potentially neurotoxic with prolonged exposure (Fig. 1). Among these, three cytokines (IL-1β, IL-2, IL-17) were found to be significantly elevated in all treatment groups. For example, exposure to MAA (p = 0.004), UIS (p < 0.001), and MAA-UIS (p < 0.001) resulted in an average of a two-to three-fold increase of IL-1β in the cortex, compared to age-matched PBS-AIR controls (see Supplementary Table Fig. 1). Similarly, it was found that there were significant increases in IL-2 in the MAA (p = 0.002), UIS (p = 0.001), and MAA-UIS (P < 0.001) groups, along with increased IL-17 in the MAA (p = 0.001), UIS (p < 0.001), and MAA-UIS (p < 0.001) groups.

For several inflammatory markers, only the presence of UIS, with or without the addition of MAA, significantly altered cytokine concentrations in the cortex. Specifically, the combined exposure of MAA-UIS increased the concentration of IL-13 (p < 0.001), IP-10 (p = 0.049), and MIP-1α (p = 0.041); exposure to UIS alone resulted in an approximately 70 pg/mL (p = 0.001), 13 pg/mL (p = 0.017), and 65 pg/mL (p = 0.004) increase in IL-13, IP-10, and MIP-1a respectively. Conversely, MAA-UIS (p = 0.041) and UIS (p = 0.030) resulted in a decrease in IL-9 concentration by 425–456 pg/mL.

For IL-1α, a significant increase in cortical concentration was observed in MAA exposure alone (p = 0.025) and in combination with UIS (p = 0.006) compared to PBS-AIR offspring, while no statistically significant differences were observed in the UIS-alone condition. Moreover, MAA (p = 0.001) and UIS (p = 0.005) alone both increased the IL-10 concentration, but this increase was absent in the MAA-UIS group (p = 0.122). Of the cytokines investigated, IL-7 was the only immune marker elevated by the MAA treatment (p = 0.030), but not UIS alone (p = 0.822) or MAA-UIS (p = 0.699). No sex-by-treatment interactions were identified in the P15 offspring.

### 3.2 P35 offspring cortex cytokines

**Figure 2:**
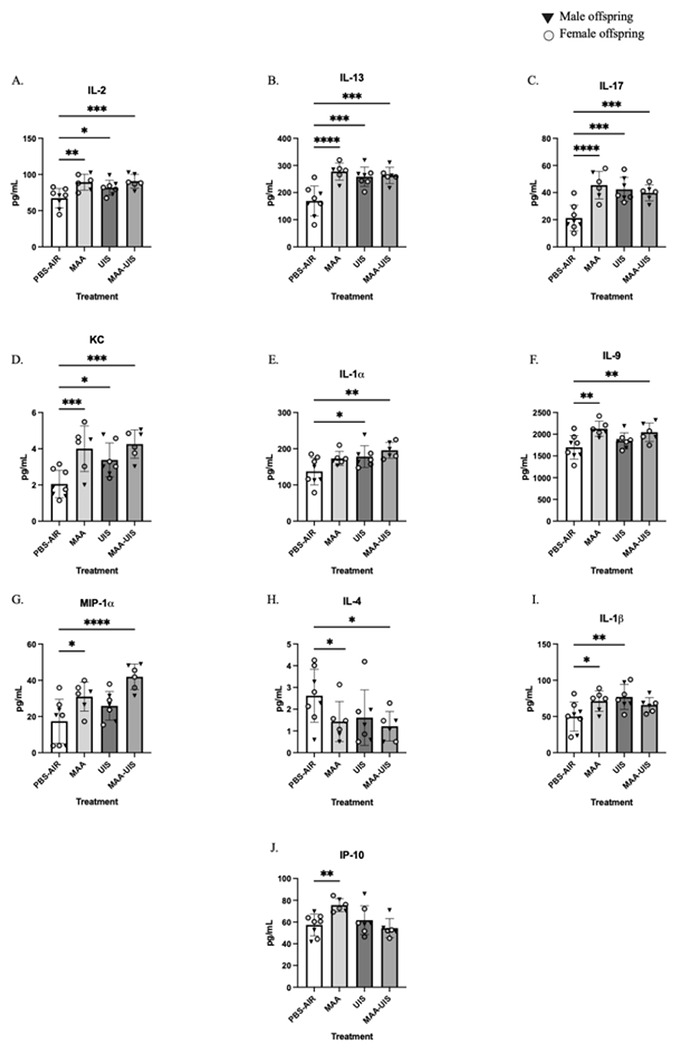
Cortical cytokine concentrations in P35 offspring brains exposed to PBS-AIR, MAA, UIS, or MAAUIS. Cytokines were assessed using multiplex bead-based immunoassays. (A) IL-2, (B) IL-13, (C) IL-17, (D) KC, (E) IL-1α, (F) IL-9, (G) MIP-1α, (H) IL-1, (I) IL-1β, and (J) IP-10 are represented as pg/mL after being normalized to total protein content. Statistical significance was determined by multilevel mixed-effects modeling.

Littermates from the P15 cytokine investigation were left undisturbed until P35, at which time brains were collected and micro-dissected into cortical and hippocampal sections. Mixed-effects models that included sex were not significantly improved over treatment-alone models for all cytokines measured (Supplementary Table 2). However, models that included treatment conditions revealed changes in several cytokines in the cortices of both sexes of MAA, UIS, and MAA-UIS offspring at P35 in a similar manner to what was identified in P15 offspring (Fig. 2). Many of the cytokine changes observed at P15 remained altered in the P35 offspring cortex. For instance, it was revealed that there was an effect of MAA, UIS, and MAA-UIS combined on IL-2 and IL-17 in P35 offspring compared to PBS-AIR control mice (IL-2: MAA p = 0.006, UIS p = 0.043, MAA-UIS p = 0.001; IL-17: MAA p < 0.001, UIS p = 0.043, MAA-UIS p = 0.001) (see Supplementary Table 2). Similar increases were also revealed for IL-13 (MAA p = 0.002, UIS p = 0.005, MAA-UIS p = 0.002) and KC (MAA p = 0.004, UIS p = 0.037, MAA-UIS p < 0.001), and a trend for IL-1β (MAA p = 0.046, UIS p = 0.008, MAA-UIS p = 0.063).

For several cytokines, the individual contribution of UIS or MAA impacted specific inflammatory profiles. For example, UIS treatment alone (p = 0.026) and in combination MAA-UIS (p = 0.002) significantly increased concentrations of IL-1α, with a trend observed in the offspring of the MAA alone condition (p = 0.064). Moreover, MAA treatment with UIS resulted in an increase in IL-9 (p = 0.018) and MIP-1α (p < 0.001), and these increases were also present in the MAA treatment alone (p = 0.015 and p = 0.033, respectively) but not in the UIS-only treated mice (p > 0.1). In addition to these MAA-induced elevations in IL-9, concomitant decreases in IL-4 concentrations were observed in mice treated with MAA (p = 0.028) and MAA-UIS (p = 0.015). IL-4 was the only cytokine investigated in P35 offspring that was revealed to be decreased due to treatment with MAA but not UIS (p = 0.088). Of all the cytokines investigated, IP-10 concentration was the only identified cytokine to be increased by one treatment only (MAA p = 0.010), with neither UIS (p = 0.435) nor MAA-UIS (p = 0.694), reaching significance.

### 3.3 P15 offspring hippocampus cytokines

**Figure 3:**
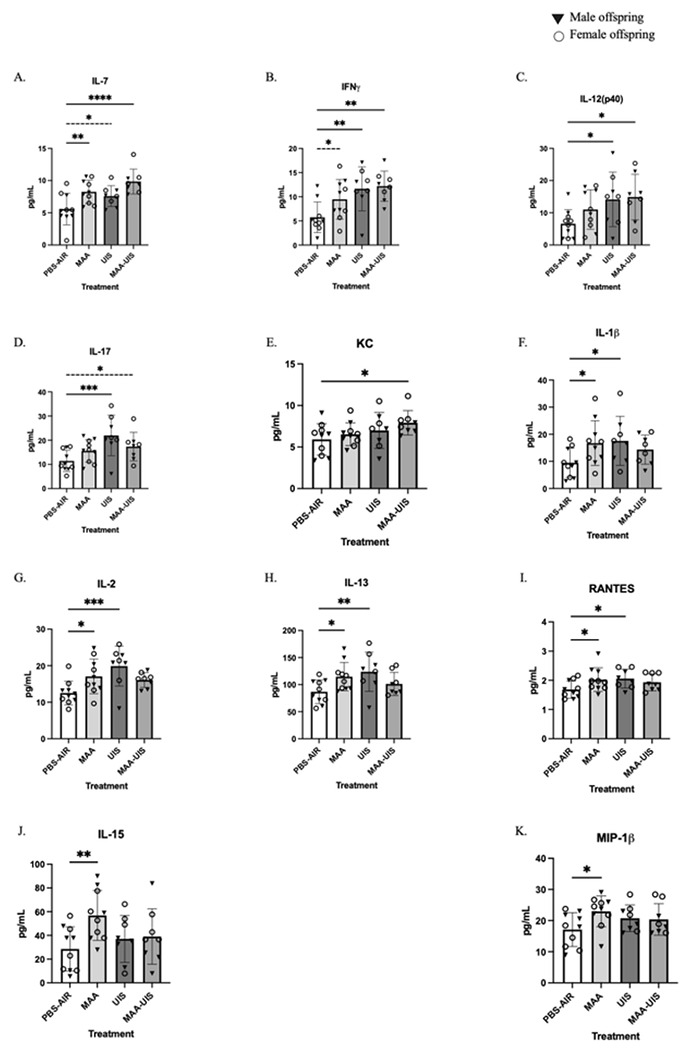
Hippocampal cytokine concentrations in P15 offspring brains exposed to PBS-AIR, MAA, UIS, or MAAUIS. Cytokines were assessed using multiplex bead-based immunoassays. (A) IL-7, (B) IFNγ, (C) IL-12(p40), (D) IL-17, (E) KC, (F) IL-1β, (G) IL-2, (H) IL-13, (I) RANTES, (J) IL-15, and (K) MIP-1β are represented as pg/mL after being normalized to total protein content. Statistical significance was determined by multilevel mixed-effects modeling.

Along with cortical sections, hippocampal homogenates from the same offspring brains were analyzed at P15 for cytokine concentrations. For all cytokines measured, the inclusion of sex in multi-level models did not significantly improve model fit (Supplementary Table 3). Similar to what was uncovered in the respective cortical sections, several inflammatory cytokines were elevated in male and female offspring of MAA, UIS, and MAA-UIS dams (Fig. 3). Interestingly, none of the cytokines investigated reached statistical significance in all three treatment groups compared to controls. However, we suspect that this may be a result of a lack of statistical power given the strong trends observed across analytes. For instance, IL-7 concentrations were significantly increased in the hippocampus of MAA (p = 0.009) and MAA-UIS (p < 0.001) offspring, while the UIS group did not reach the statistical threshold for significance (p = 0.051). In a similar manner, IFNγ was revealed to be elevated by UIS (p = 0.008) and MAA-UIS (p = 0.004), with similar elevations observed the MAA group but not reaching statistical significance (p = 0.056). MAA-UIS exposure was also revealed to have a treatment effect on IL-12(p40) (p = 0.028), as did UIS alone (p = 0.028), but these increases were not present in the hippocampus of offspring born from the MAA treatment alone (p = 0.151). Concentrations of IL-17 in the offspring hippocampus were elevated at P15 in response to maternal UIS exposure alone (p = 0.002) and in combination MAA-UIS (p = 0.051). However, these increase in IL-17 were not detected in the hippocampus of MAA-only offspring (p = 0.135).

The chemokine CXCL1, referred to as KC, was only observed to be increased in the hippocampus of offspring who were exposed to the combination of MAA-UIS (p = 0.044), but not MAA alone (p = 0.480) or UIS alone (p = 0.254), suggesting a potentially additive effect of the two environmental exposures. In contrast, several cytokines were elevated by MAA and UIS alone compared to the PBS-AIR control offspring, but these elevations did not reach statistical significance in the combined MAA-UIS condition. These increases included IL-1β (MAA p = 0.047, UIS p = 0.038), IL-2 (MAA p = 0.026, UIS p = 0.002), IL-13 (MAA p = 0.034, UIS p = 0.011), and RANTES (MAA p = 0.041, UIS p 0.034). Additionally, two cytokines were found to be impacted by MAA alone compared to the control, and not the UIS or MAA-UIS treatment groups. Specifically, IL-15 (p = 0.009) and MIP-1β (p = 0.028) were found to be elevated by MAA, but not UIS or MAA-UIS.

### 3.4 P35 offspring hippocampus cytokines

Hippocampal homogenates from P35 offspring were also assessed for cytokine differences; however, multilevel mixed-effects modeling did not reveal any significant effects of treatment or sex in on the concentration of all cytokines measured (p > 0.05; see Supplementary Materials).

### 3.5 P15 Microglia density

**Figure 4:**
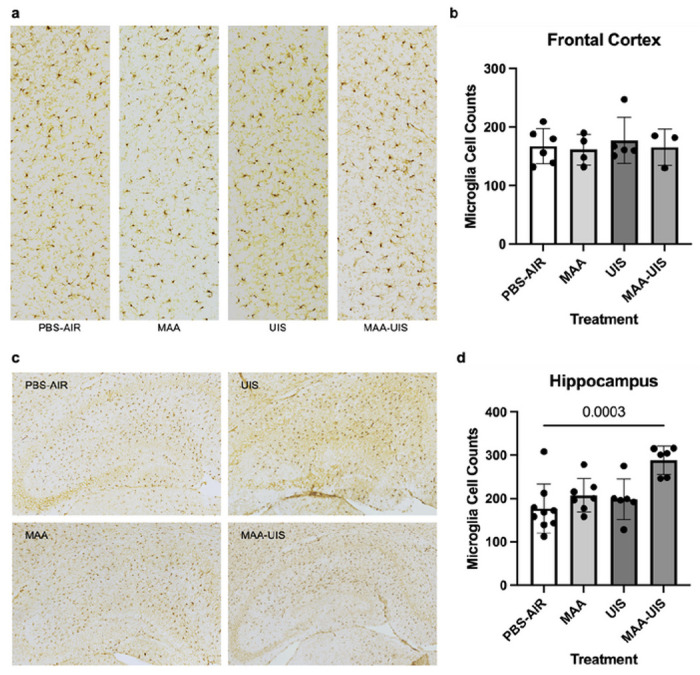
Microglia density in the hippocampus and frontal cortex of MAA, UIS, and MAA-UIS of P15 offspring. (a and c) Staining for IBA-1 with diaminobenzidine (DAB) was used to label microglia in coronal sections of p15 mice in the (a) frontal cortex and (b) hippocampus. (b and d) Quantification of microglial density in the (c) frontal cortex and (d) hippocampus. Statistical significance determined via one-way ANOVA. In the frontal cortex, n=6 (PBS-AIR), n=4 (MAA), n=5 (UIS), n=3 (MAA-UIS). In the hippocampus, n=9 (PBS-AIR), n=7 (MAA), n=6 (UIS), n=6 (MAA-UIS).

Microglia within a 554.7 μm by 1,232.1 μm box spanning the anterior cingulate cortex, prelimbic area, and infralimbic area were counted using ImageJ by an investigator blinded to treatment condition. Analysis of microglia density in the dentate gyrus, CA1, CA2, and CA3 regions of the hippocampus in p15 mice showed significant differences between the PBS-air group and the MAA-UIS group (*p* = 0.0166; Fig. 4). There were no significant changes in the microglia density between treatment groups in the frontal cortex of p15 mice (Fig. 4). Additionally, in both the hippocampus and cortex, no differences in microglia density were seen in the single treatment groups (MAA or UIS) when compared to the PBS group.

## 4. DISCUSSION

Among the many well established environmental factors that can impact fetal neurodevelopment, asthma and air pollution represent two major sources of immune stimulation that are on the rise, making them a significant concern for pregnant individuals. Based on previous studies of maternal immune activation with asthma, and PM exposure during pregnancy, we hypothesized that the combination of these two environmental stimuli would result in an exacerbated neuroimmune response in offspring. Although the appearance of an additive or synergistic effect of MAA and UIS exposure combined was limited, we did identify increases in cytokine concentrations across all treatment groups in the cortex and hippocampus that may suggest converging pathways for each insult/exposure. Importantly, some of these elevations appear to be sustained across treatment groups from adolescence into early adulthood in the cortex, demonstrating lasting impacts of these gestational exposures on the neuroimmune environment later in life. Although we identified increases in cytokines in the hippocampus within all treatment groups at P15, these did not remain elevated into early adulthood. Overall, these data show that MAA and UIS environmental stimuli can result in an altered neuroimmune environment that persists from juvenile to adult timepoints.

The allergic response in the lungs of individuals with asthma is characterized by an influx of immune cells, such as neutrophils, mast cells, macrophages, and T-helper (T_H_)2 cells. Our mouse model of MAA previously showed elevated IL-4, IL-5, IL-17, and IFNγ in the lung and peripheral blood of mice exposed to aerosolized OVA during pregnancy (Schwartzer et al., 2017; Church et al., 2021; Tamayo et al., 2022). These increases in maternal serum cytokines result in neuro-immune signaling changes in the fetal brain during in utero development (Tamayo et al., 2022). Our present data extend these findings by revealing evidence of increases in cortical and hippocampal cytokines in juvenile mice of MAA dams. In the cortex, for example, MAA alone increased IL-1β, IL-2, IL-17, IL-1α, IL-10, and IL-17. In the hippocampus, we identified IFNγ, IL-1β, IL-2, IL-7, IL-13, IL-15, MIP-1β, and RANTES as being elevated in P15 offspring of MAA-exposed dams. These observed increases in MAA compared to PBS-AIR controls demonstrates the independent neurodevelopmental impact of allergic inflammation during pregnancy on offspring neuroinflammation. In addition to these findings in the MAA only treatment group, we also identified the UIS treatment (in the absence of MAA) resulting in an increase of cytokine concentration in juvenile offspring, specifically, in IL-1β, IL-2, IL-13, IL-17, IP-10, MIP-1α, and IL-10. Moreover, increases in cytokines as a result of UIS exposure alone were also identified in the hippocampus of juvenile offspring, with elevated IFNγ, IL-1β, IL-2, IL-7, IL-12(p40), IL-13, IL-17, and RANTES. To the authors’ knowledge, investigations into these neurobiological outcomes in offspring under UIS exposure during gestation have not been previously reported, highlighting the novelty of our model and findings.

In addition to the independent effects of MAA or UIS treatment on cytokine concentrations in the cortex and hippocampus of juvenile offspring, these elevations were most often coupled with elevations in the MAA-UIS combined treatment group. Most notably, we observed elevations in the cortex of IL-1β, IL-2, IL-13, and IL-17 in dual-exposed MAA-UIS offspring. These cytokines were also trending or increased in the MAA and UIS single treatment groups as well as the MAA-UIS treatment group at P15, and they remained elevated into the P35 timepoint. Our data suggest a sustained elevation in these four cytokines from P15 to P35 as a result of both MAA and UIS that have the potential for long-lasting impacts on neurodevelopment in the cortex of these offspring.

Consistent with the pleiotropic nature of cytokines in the central nervous system (CNS), IL-1β, IL-2, IL-13, and IL-17 have all been identified as having neurotrophic properties (Awatsuji et al., 1993; De Araujo et al., 2009; Park et al., 2018; Lock et al., 2002; Kebir et al., 2007; Milovanovic et al., 2020; Ma et al., 2014; Rochman et al., 2013; Brombacher et al., 2017). Indeed, high concentrations (500 ng/mL) of IL-1β can have neurotoxic effects on neurons when exposed for 3–5 days (Park et al., 2018), and IL-17 is detected at high levels in the CNS in multiple sclerosis and associated with the neuroinflammatory pathology of the disease (Lock et al., 2002; Kebir et al., 2007). Compared to these neurotoxic concentrations, our data represent moderate increases in cytokines with less than 2.5-fold increases in treatment groups compared to PBS-AIR controls, and may not represent overt inflammation per se. However, these smaller changes in brain cytokine levels during the juvenile period may be biologically significant given their alternative functions in promoting neuronal survival and neurodevelopment. For example, IL-1β acts as a chemokine that guides neurite outgrowth in cortical neurons (Ma et al., 2014), and IL-17 acts in initiating the release of brain-derived neurotrophic factor (BDNF), glia-derived neurotrophic factor (GDNF), and nerve growth factors (NGF) associated with neuronal cell survival and repair (Milovanovic et al., 2020). Taken from this view, these cytokines, which are generally considered overtly inflammatory, may be having a more subtle impact on the neuroarchitecture of offspring brains than models finding dramatic increases in concentration of IL-1β and IL-17.

Further demonstrating the neuropoietic nature of these cytokines within the CNS and adding to the idea that the moderate increases observed in this model may be altering the neuropatterning of the offspring brains, IL-2 has been found to have neurotrophic properties and is necessary for proper cytoarchitecture in development (Beck et al., 2005; Beck et al., 2005). In addition, IL-13 is often considered anti-inflammatory in the CNS, with some studies pointing to a neuroprotective impact in CNS diseases and injuries (Miao et al., 2020; Guglielmetti et al., 2016; Le Blon et al., 2016; Pan et al., 2013). Both IL-2 and IL-13 are among several cytokines that are known to decrease in concentration at P14 under homeostatic conditions, and this developmental timepoint in mice is characterized by a high degree of synaptogenesis and pruning (Garay et al., 2013; Morato Torres et al., 2020). In contrast, our model, which investigated cytokine concentrations at P15, still within this window of high synaptogenesis, showed increased IL-2 and IL-13, representing a shift in homeostatic load. Taken together, it may be that these sustained moderate increases in cytokines of the cortex are changing the trajectory of cortical development and promoting altered connectivity in the cortex linked to behavioral changes such as decreased social interaction and repetitive behaviors previously identified in our model (Schwartzer et al., 2015; Church et al., 2020). This phenomenon of altered connectivity has also been implicated in the core behaviors associated with ASD, specifically the social deficits and restrictive and repetitive behaviors (Conti et al., 2017; Maximo et al., 2014; Mills et al., 2016).

Similar to our findings in the cortex, we also identified elevations in several cytokines, most notably IFNγ and IL-7, at P15 in the hippocampus of MAA alone, in UIS alone, and MAA-UIS offspring compared to controls. IFNγ receptors are present on both neurons and glia (Ottum et al., 2015). In the hippocampus, IFNγ appears to play a role in synaptogenesis (Lee et al., 2006). Some investigators have found that overexpression resulted in increased neurogenesis in the dentate gyrus, and because of its neuromodulatory effects, it has been suggested that this may impact cognition and social behavior as a result (Flood et al., 2019; Filiano et al., 2016; Baron et al., 2008). Additionally, IL-7 promotes survival and neurite outgrowth in hippocampal neurons (Macia et al., 2010; Michaelson et al., 1996). Considering the effects of IL-7 and IFNγ, and that the hippocampus is a major neurogenic niche in the developing brain, future studies may benefit from investigating the potential for hippocampal overgrowth in offspring brains in response to UIS or allergic asthma inflammation during pregnancy. Indeed, this phenomenon of hippocampal overgrowth has been identified in cases of ASD (Groen et al., 2010; Murphy et al., 2012, Rojas et al., 2006) and has been implicated in the deficits associated with emotion perception and sensory processing in ASD individuals (Groen et al., 2010, Banker et al., 2021). Curiously, the observed increases in hippocampal cytokine concentrations at P15 were not observed in any treatment group of P35 offspring compared to PBS-AIR controls. Although we can only speculate about these findings, it may be that these changes resolve during adolescence when additional brain maturation may be present to compensate for developmental overgrowth much in the same way that volumetric increases in the hippocampus of ASD individuals do not persist into adulthood (Groen et al., 2010; Rojas et al., 2006). Although IL-7 and IFNγ are only two examples of cytokines that we found elevated among the treatment groups, they illustrate the broader findings that treatment with MAA, UIS, or MAA-UIS can alter the hippocampal neuroimmune environment with potential consequences to behavioral outcomes.

Prenatal insults have been shown to have lifelong impacts on microglia function and are suspected to play a prominent role in neurodevelopmental disorders (Vogel Ciernia et al., 2018; Eggen et al., 2013; Knuesel et al., 2014; Slusarczyk et al., 2015; Giovanoli et al., 2016; Hughes et al. 2023). In ASD, some postmortem studies have identified differences in microglia density and morphology in brains of individuals (Koyama et al., 2015; Morgan et al., 2010; Morgan et al., 2014). In our previous study of MAA, we found DNA methylation differences in adult microglia, and several of these changes occurred in regulatory genes that are shared among some ASD individuals (Vogel Ciernia et al., 2018). Given these findings, we sought to examine the density of microglia in the P15 brains of our MAA and UIS exposure model. We observed a significant increase in microglia density within the hippocampus of offspring exposed to MAA-UIS, but these increases were not present in the frontal cortex. One plausible explanation for why these increases were only observed in the hippocampus may be due to the higher density of microglia known to be present in the hippocampus. This higher density of microglia is thought to make the hippocampus more vulnerable to inflammation (Réus et al., 2015; Choi et al., 2011), and disruptions in the dentate gyrus have been linked to neurodevelopmental disorders (Cai et al., 2018; Yagishita et al., 2017). Our findings of increased microglia density in the hippocampus of MAA-UIS offspring mirror data from another maternal immune activation model that utilizes the viral mimic poly(I:C). Specifically, Juckel et al. (2011) reported an increase in microglia density in the hippocampus but not the cortex of offspring born from immune-activated dams. Similarly, another study of maternal immune activation using LPS stimulation showed an increase in microglia density in the hippocampus (Diz-Chaves et al., 2013). While it is difficult to make conclusions about microglial function based on density data alone, our observed difference in the hippocampus in combination with similar reports from other maternal immune activation models (Juckel et al., 2011; Diz-Chaves et al., 2013) suggest that asthma allergy and PM mediated immune activation during pregnancy can result in a deviation from homeostatic activity in the offspring hippocampus.

Although we did not collect maternal data in this preliminary study, data from previous MAA studies demonstrates increased systemic inflammation characteristic of an allergic asthma response, specifically with increased IL-4, IL-5, and IL-13 (Schwartzer et al., 2017; Church et al., 2020; Tamayo et al., 2022), suggesting the potential for a similar response in dams of MAA in the current model. Speculation about the systemic impacts of UIS on the maternal immune system, however, is difficult. Many studies of PM exposure suggest IL-6, IL-8, and TNFα as the main cytokines upregulated in response to PM exposure (Mitschik et al., 2008; Silbajoris et al., 2011; Musah et al., 2012; Gong et al., 2022; Longhin et al., 2018). This difference in cytokine response highlights the potential reason we see differences in the impact between MAA and UIS in our model. However, models of PM can vary widely in the size of PM and composition (Mitschik et al., 2008), making speculation about the maternal response in the UIS groups, and the potential role this plays in offspring neurodevelopment, difficult. This variation in PM studies underscores the need for future investigations to identify the maternal cytokine milieu in this model.

While our findings do not necessarily demonstrate an additive effect of MAA and UIS with regard to the cytokines we investigated, we did see a synergistic impact of MAA-UIS on microglia density in the hippocampus. These findings demonstrate the potential for additive effects of maternal asthma exposure when coupled with PM exposure. Independently, studies have shown in both humans and animal models that PM exposure during pregnancy can increase the susceptibility of offspring developing asthma (Mortimer et al., 2008; Wang et al., 2013; Hua et al., 2023). This increased susceptibility of asthma in offspring is also seen in children of asthmatic mothers (Martel et al., 2009; Mattes et al., 2013; Murphy 2022), suggesting the potential for systemic immune disfunction when these two stimuli are combined during pregnancy. The findings in this unique model of MAA and UIS exposure highlight the importance of investigating the impact of these closely linked and prevalent environmental factors.

## 5. CONCLUSIONS

Our data add to our previous studies on the impact of MAA on fetal brain development, showing here that this model impacts region-specific cytokine concentrations in both the juvenile and adolescent periods. To the investigators’ knowledge, this was the first study to assess the impact of ultrafine iron-soot exposure during gestation on offspring neurobiology. Moreover, our identification of regional changes in cytokine concentrations implicate a potential driving force behind our previously identified behavioral changes in the MAA model. Taken together, these data highlight the importance of understanding the impact that common environmental stimuli can have on fetal development, and the potential for these stimuli to have long lasting changes in offspring.

## Data Availability

The datasets used and/or analyzed during the current study are available from the corresponding author on reasonable request.

## Abbreviations

ASD: Autism spectrum disorders
PM: Particulate matter
MAA: maternal allergic asthma
UIS: ultrafine iron-soot
OVA: ovalbumin
GD2: gestational day 2
PBS: phosphate buffered saline
P: postnatal days
IL: interleukin
IFNγ: interferon gamma
MAA-UIS: MAA and UIS exposure
LPS: lipopolysaccharide
Poly(I:C): polyinosinic:polycytidylic acid
ADHD: attention deficit, hyperactive disorder
IS: iron-soot
NGS: normal goat serum
DAB: 3,3’-diaminobenzidine
G-CSF: granulocyte colony stimulating factor
GM-CSF: granulocyte colony stimulating factor
IP-10: interferon gamma-induced protein 10
KC: keratinocyte chemoattractant
MCP-1: monocyte chemoattractant protein-1
MIP-1α: macrophage inflammatory protein-1 alpha
RANTES: chemokine ligand 5
TNF-α: tumor necrosis factor alpha
AIC: Akaike Information Criterion
T_H_: T-helper
CNS: central nervous system
BDNF: brain-derived neurotrophic factor
GDNF: glia-derived neurotrophic factor
NGF: nerve growth factors
MIA: maternal immune activation
